# Percutaneous Management of a Long Saphenous Vein Graft Aneurysm: A Case Report and Review of Literature

**DOI:** 10.4061/2009/981292

**Published:** 2009-09-02

**Authors:** Carmelo J. Panetta, Williaim Schneider, Max A. Boller

**Affiliations:** Park Nicollet Heart and Vascular Center, 6500 Excelsior Blvd, St. Louis Park, MN 55426, USA

## Abstract

Aneurysms of saphenous vein grafts are rare but can result in complications such as myocardial infarction or death. Percutaneous treatment has included a variety of approaches, including covered stents. Long aneurysms in saphenous vein grafts pose an additional challenge due to the lack of coronary covered stents with sufficient length. We present successful treatment of a long saphenous vein graft aneurysm with use of peripheral covered stents over two coronary guidewires, a 55-centimeter 8-French sheath and no guide catheter.

## 1. Introduction

**Figure 1:**
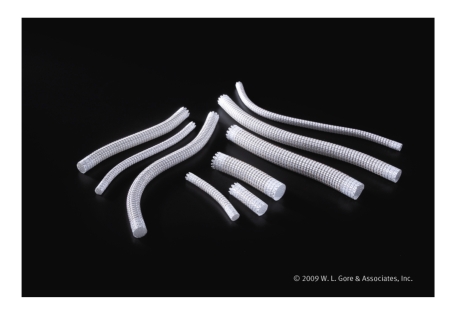
Viabahn PET covered stents. Picture provided by W. L. Gore and Associates.

Saphenous vein graft (SVG) aneurysm is a rare complication presenting in a variety of ways including an abnormality on chest X-ray, myocardial ischemia, or graft rupture [1–3]. Surgical revision is often recommended but often carries a high risk [3]. Percutaneous approaches have included covered stents, use of a vascular plug, or coiling of the aneurysm [4–13]. These approaches are successful for sacular aneurysms, but fusiform aneurysms can pose a problem due to limited length of coronary covered stents. One successful approach was the use of a long Wallstent to act as a scaffold in the aneurysm and then deploy multiple coronary covered stents within the Wallstent [13]. We report the successful coverage of a fusiform saphenous vein graft aneurysm with use of peripheral Viabahn covered stents (Figure 1) over two coronary guidewires for adequate support via a 55-centimeter (cm) 8-French sheath without a guide catheter.

## 2. Case Report

### 2.1. History

**Figure 2:**
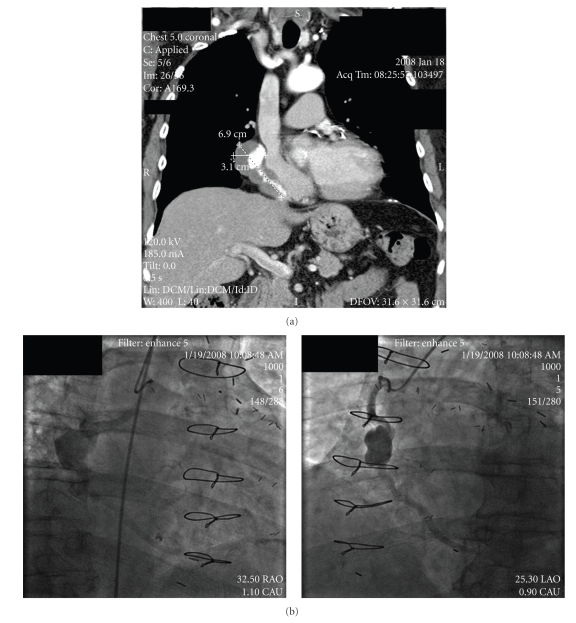
(a) Computer tomography (CT) in January 2008 of chest revealing a 3.1 cm × 6.9 cm aneurysm with contrast filling the inner lumen, and possible thrombus filling the remaining space within the aneurysm. (b) Coronary angiography depicting the aneurysm of the mid SVG, with TIMI 2 flow in right posterior descending artery (PDA) in both right anterior oblique (RAO) and left anterior oblique (LAO) views.

The patient is a 74-year-old man with history of coronary artery bypass surgery in 1988 with a known ectatic and degenerative SVG to the right posterior descending coronary artery (PDA). He presented in January 2008 with progressive right-sided pleuritic chest pain and peripheral edema. A chest X-ray revealed a rounded mass 3.7 cm in diameter along with right boarder. Follow up computer tomography (CT) scan of the chest revealed a mass worrisome for an SVG aneurysm estimated 3.1 cm in the proximal-mid section, extending approximately 6.9 cm distally and compressing the right atrium (Figure 2(a)). The patient was referred to our institution, and coronary angiography confirmed an aneurysm (Figure 2(b)) in the SVG with TIMI 2 flow to the right PDA. A discussion was held with cardiothoracic surgery as well as several staff members on several options. Coronary artery bypass surgery was felt too high risk given history of thrombocytopenia, chronic kidney disease as well as the enlarging aneurysm in setting of a second open heart operation. Coiling the aneurysm was not practical due to the fusiform nature of the aneurysm.

### 2.2. Procedure

**Figure 3:**
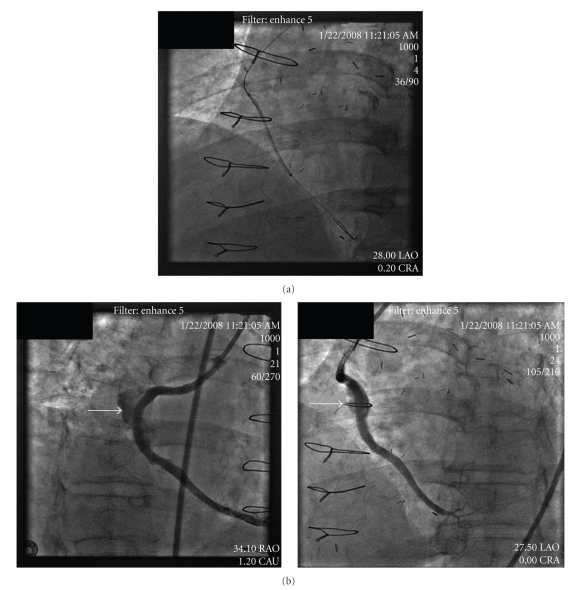
(a) Placement of the Viabahn self expanding stent with two guidewires in distal SVG and no guide catheter. (b) Coronary angiography after placement of the stents with residual contrast entrapped in the aneurysm (arrows) in both RAO and LAO views with TIMI 3 flow in PDA.

A percutaneous approach was chosen with use of a Viabahn self expanding nitinol polyethylene terephthalate (PET) covered stents (W. L. Gore and Associates, Newark, DE). Viabahn PET covered stents were considered to have sufficient length to cover the aneurysm and have been proven successful in treatment of peripheral arterial aneurysms [14]. The stents typically are delivered via only the 7- or 8-French sheath without use of a guide catheter and passed over the large 0.035 inch wire for support. As the inner lumen of an 8-F sheath is larger than an 8-F guide, the use of a larger guide could increase the risk for bleeding in the femoral artery access site. In addition, the use of a 0.035-inch guide wire could be more challenging to place in the coronary artery downstream of the aneurysm due to risk for embolization of debris from the aneurysm. To accommodate, we used a long 55 cm length 8-French Brite Tip sheath (Cordis, Johnson and Johnson, Miami Lakes, Fl) rather than a larger guide catheter but lost the capability of directly injecting contrast in the SVG to properly visualize the placement of the stents. A 5 mm × 5 cm followed by a 5 mm × 2.5 cm covered stents were delivered over two wires, an Asahi Prowater coronary guide wire (Abbot Laboratories, Abbott Park, IL) and an Ironman coronary guide wire (Abbot Laboratories, Abbott Park, IL) rather than one large 0.035 inch guide wire (Figure 3(a)). We considered using a distal protection device, but were unable to pass the filter wire without concern for embolization of debris from within the aneurysm. There was a small section of the aneurysm not covered by the two Viabahn PET covered stents and a 4 mm × 16 mm Jostent GraftMaster polytetraflouroethylene (PTFE) covered stent (Abbot Laboratories, Abbott Park, IL). A 4.5 mm × 16 mm Liberte bare metal stent (Boston Scientific, Maple Grove, MN) was deployed to overlap the Viabahn stent and post dilated with Mavrick XL 5.5 mm × 15 mm balloon (Boston Scientific, Maple Grove, MN). Final images revealed entrapment of contrast in the aneurysm and TIMI 3 flow in PDA (Figure 3(b)). The patient had an uneventful hospital course and was discharged with no further chest pain.

### 2.3. Follow-Up

**Figure 4:**
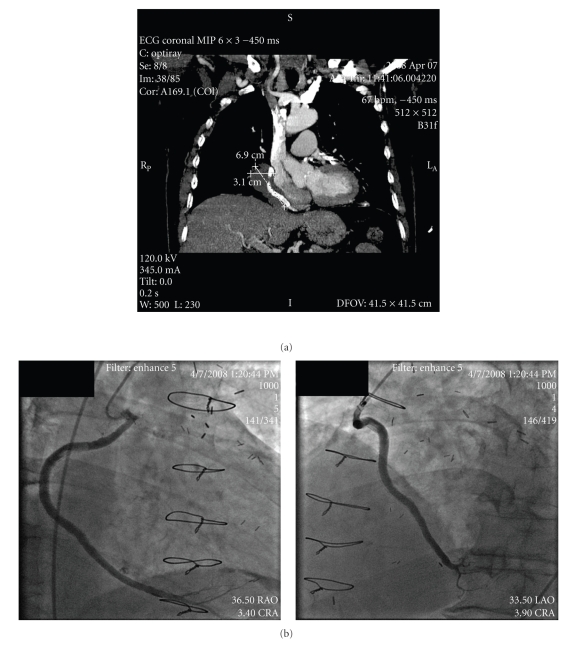
(a) CT of the chest in April 2008 revealing aneurysm size (3.1 cm × 6.9 cm) has remained unchanged. (b) Coronary angiography in April 2008 in both RAO and LAO views revealing patent stents and no evidence of leak through the covered stents into aneurysm.

He returned in April 2008 for follow-up CT scan and coronary angiography and reported being pain free and edema had not returned. CT scan revealed that the aneurysm remained unchanged at 3.1 cm × 6.9 cm (Figure 4(a)) in size. No contrast was visualized outside the lumen, and no severe lesions were seen within the covered stents (Figure 4(b)). At one year he remained asymptomatic on clinical follow-up.

## 3. Discussion

Coronary aneurysms are defined as a localized dilation of 1.5 times the diameter of the adjacent segment [15]. A fusiform aneurysm is twice as long as it is wide versus the saccular, which is larger in width than length. An ectatic coronary artery is defined as diffusely dilated greater than 50% of arterial diameter [15]. Etiology of aneurysms of vein grafts is not known but related to atherosclerosis, thrombosis, and systolic and diastolic bidirectional blood flow which all could create and rapidly enlarge aneurysms [6, 16]. Unfortunately, some enlarge and cause pain, rupture with possible fistula formation, myocardial ischemia or compress adjacent structures [2, 3]. This patient had both chest pain and compression of adjacent structures by CT imaging. He had no evidence for ischemia except for slow flow in the right PDA. The aneurysm was large but not considered giant as it was not greater than 4 cm in diameter [3]. The aneurysm was both saccular in appearance in the proximal-mid section and fusiform in the mid to distal section of the graft (Figure 2(a)). The SVG placed twenty years earlier had functioned well, documented by angiography four years earlier, although appearing ectatic. Surgery would have been preferred although one series reported no difference in mortality on a retrospective analysis [17]. The authors discuss a potential algorithm for management. In the setting of symptoms, low operative risk and viable myocardium endovascular repair or surgery is reasonable. In the setting of high operative risk, endovascular exclusion/coiling with or without percutaneous revascularization is reasonable. For this patient, reoperation has inherent risks; in addition, if the graft is not completely removed, a second aneurysm may arise [3].

Several reports on transcatheter coil embolization have been effective for saccular aneurysms [4, 5]. An amplatzer plug has been used effectively to occlude a giant saccular SVG aneurysm [6]. Use of a covered endograft in the ascending aorta was used to occlude an SVG that had closed distal to an enlarging aneurysm [7]. A vein covered stent was successful in covering a saccular aneurysm in a saphenous vein graft [8]. Several reports with PTFE covered stents have been successful, including out to one year [9–13], although a large false aneurysm was unsuccessfully covered by a PTFE-coated stent [14]. A long aneurysm was ingeniously covered with self expanding Wallstent initially as a scaffold, followed by four PTFE covered stents; with the only caveat at six months there was a residual leak that required an additional PTFE covered stent [13]. We were able to cover the aneurysm with two Viabahn PET covered self expanding stents and one coronary PTFE covered stent. An important limitation of this technique is lack of a guide catheter for adequate viewing of the SVG aneurysm with contrast while placing the peripheral stents, which we believe contributed to the need for the additional coronary covered stent. In view of this limitation, we believe that this approach should not be considered routine for management of SVG aneurysms.

## 4. Conclusions

We report the successful coverage of this patient's long SVG aneurysm with use of Viabahn self expanding nitinol covered stents over two wires via a 55 cm sheath. His outcome was stable by coronary angiography and CT scan at 4-month postprocedure as well as clinical follow-up after the intervention. We presume potential exists for further aneurysm formation at the proximal and distal regions of the SVG not covered by the stent. His medical therapy includes antiplatelet agents as well as plaque stability with HMG-CoA reductase inhibitors.
